# Equity improvements in maternal and newborn care indicators: results from the Bardiya district of Nepal

**DOI:** 10.1093/heapol/czv077

**Published:** 2015-08-24

**Authors:** Bareng AS Nonyane, Ashish KC, Jennifer A. Callaghan-Koru, Tanya Guenther, Debora Sitrin, Uzma Syed, Yasho V Pradhan, Neena Khadka, Rashed Shah, Abdullah H Baqui

**Affiliations:** ^1^International Center for Maternal and Newborn Health, Johns Hopkins Bloomberg School of Public Health, Baltimore, MD, USA,; ^2^Department of Women and Children, Uppsala University, Uppsala, Sweden,; ^3^Save the Children, Washington, DC, USA and; ^4^Society of Public Health Physician

**Keywords:** Community-based interventions, equity, maternal care, newborn care

## Abstract

Community-based maternal and newborn care interventions have been shown to improve neonatal survival and other key health indicators. It is important to evaluate whether the improvement in health indicators is accompanied by a parallel increase in the equitable distribution of the intervention activities, and the uptake of healthy newborn care practices. We present an analysis of equity improvements after the implementation of a Community Based Newborn Care Package (CB-NCP) in the Bardiya district of Nepal. The package was implemented alongside other programs that were already in place within the district. We present changes in concentration indices (CIndices) as measures of changes in equity, as well as percentage changes in coverage, between baseline and endline. The CIndices were derived from wealth scores that were based on household assets, and they were compared using *t*-tests. We observed statistically significant improvements in equity for facility delivery [CIndex: −0.15 (−0.24, −0.06)], knowledge of at least three newborn danger signs [−0.026(−0.06, −0.003)], breastfeeding within 1 h [−0.05(−0.11, −0.0001)], at least one antenatal visit with a skilled provider [−0.25(−0.04, −0.01)], at least four antenatal visits from any provider [−0.15(−0.19, −0.10)] and birth preparedness [−0.09(−0.12, −0.06)]. The largest increases in practices were observed for facility delivery (50%), immediate drying (34%) and delayed bathing (29%). These results and those of similar studies are evidence that community-based interventions delivered by female community health volunteers can be instrumental in improving equity in levels of facility delivery and other newborn care behaviours. We recommend that equity be evaluated in other similar settings within Nepal in order to determine if similar results are observed.

**Key Messages**

- The results of our analysis indicate that that the combination of the Community Based Newborn Care Package and other programs in the Bardiya district of Bangladesh resulted in statistically significant improvements in equity for facility delivery, knowledge of at least three newborn danger signs, breastfeeding within 1 h, at least one antenatal visit with a skilled provider, at least four antenatal visits from any provider and birth preparedness.
- We recommend that these programs be continually evaluated and improved in order to make it feasible for them to be scaled up to the national level. We also recommend that equity be evaluated in other settings within Nepal in order to determine if there is improvement in those areas as well.

## Introduction

The latest statistics indicate that the decline in neonatal mortality has been slower than that of the overall under-5 mortality, and in 2013 there were 2.9 million neonatal deaths, making up 44% of all under-5 deaths (Lawn *et al*. 2014). Earlier studies have shown a similar trend (Black *et al*. 2003, 2010; Lozano *et al.* 2011; Liu *et al*. 2012; IGME 2013, Oestergaard *et al*. 2013). Low- and middle-income countries in Sub-Saharan Africa and Asia have high inequality in access to healthcare, and these countries carry most of the burden of neonatal deaths (Braveman and Gruskin 2003; Sastry 2004; Lawn *et al*. 2005, 2014). Within countries, neonatal mortality rates are higher in poor households than in less poor households (Fenn *et al*. 2007; McKinnon *et al*. 2014).

In the last 15 years, reducing maternal and child deaths has been made a priority as one of the Millennium Development Goals (MDGs) and interventions that are tailored to suit local contexts have been devised and implemented (Bhutta *et al*. 2005; Darmstadt *et al*. 2005b; Baqui *et al.* 2008b; Kc *et al*. 2011a). Success of these interventions is dependent on whether they reach people of different income levels in an equitable way. Victora *et al.* (2012) conducted a review of such interventions in 35 countries and found that the quicker the rise in coverage of the interventions, the more equitable they were. Victora *et al.* also noted the importance of accounting for equity when programs are adopted for scale up.

Equity is an increasingly important part of the post-MDG agenda for child health (World Health Organization 2012; Bryce *et al*. 2013). Coverage increases that don’t reach the poorest do less to reduce mortality and models suggest that scaling up interventions among the poorest households saves more lives than scaling up only among the richest households (Ruhago *et al*. 2012).

### Inequalities in access to healthcare and neonatal mortality in Nepal

Nepal is ranked 157 out of 187 countries in terms of human development, and 25% of the people live below the poverty line (UNDP 2013). The majority of the poor are women, Dalit and disadvantaged Janjati (indigenous groups). According to the Central Bureau of Statistics (CBS 2011), the most disadvantaged are households from the Far Western region and other remote hill and mountain areas, as well as the Terai Adibasi (indigenous community) in the plains. These groups have limited access to quality health services. There is also a big gap in neonatal mortality rates between dis-advantaged and non-disadvantaged caste and ethnic groups (Pandey *et al.* 2013; Paudel *et al.* 2013).

Over the last 15 years, the under-5 mortality rate declined by 54%, going from 118 to 54 deaths per 1000 live births (MoHP 2001, 2006, 2011, Kc *et al*. 2011a). However, improvements in neonatal health have not kept up with this pace. The neonatal mortality declined by only 34 percent during the same period, going from 50 per 1000 live births in 1991–1995 to 33 in 2006–2010. Early neonatal deaths accounted for 85% of all neonatal deaths in Nepal in the year 2013 (Paudel *et al.* 2013).

An analysis using 2001 data found that if the neonatal mortality rate for all households in Nepal was reduced to that of the least poor quintile, overall neonatal mortality would reduce by almost 40% (Fenn *et al*. 2007). McKinnon *et al*. (2014) found that the neonatal mortality rate was 70% lower among the least poor households compared with the poorest households, and that wealth inequalities in neonatal mortality had not reduced significantly along with the reduction in the overall neonatal mortality rates.

Skilled birth attendance (SBA) is one of the least equitably distributed interventions (Barros *et al*. 2012b), but also one of the most important for newborn survival (Darmstadt *et al*. 2005a). In an analysis of 54 low-income countries, Nepal ranked 10th highest inequality in SBA coverage (Darmstadt *et al*. 2005a).

A study by Nguyen *et al.* (2013) investigated the change in under-5 mortality across five equity markers in Nepal, namely, urban vs rural location; ecological region (mountain, hill or plain); development region; caste or ethnicity; and wealth scores based on assets location. They found a non-statistically significant decline in absolute inequalities, while relative inequalities across equity markers have remained stable. They projected that by 2015, neonatal deaths would account for 65% of all under-5 mortality.

### Nepal maternal and child-care interventions

Nepal has achieved high reductions in overall child mortality, due in part to national policies and efforts to increase coverage of maternal and child-care interventions through a cadre of Female Community Health Volunteer (FCHV) programme (Government of Nepal 2006; Ministry of Health and Population 2010; Pradhan *et al*. 2012).

In order to reduce the social inequities and improve health behaviour, the Government of Nepal’s Ministry of Health and Population (MoHP) developed the Nepal Health Sector Programme and Gender Equality and Social Inclusion strategy, as well as other initiatives to improve maternal and neonatal health. These programs supported peripheral level health facilities, community health workers (CHWs), FCHVs and the mother’s group (MG) for maternal and child health programs (SNL 2002; Government of Nepal 2006; Era 2009; Ministry of Health and Population 2010; UNICEF WHO 2012). FCHVs are community-based health care volunteers who are selected from within their communities by the MG committees. CHWs are a cadre of community-based government-paid workers who provide preventative and curative care, family planning, maternal and child health services as well as supervisory support to FCHVs. Despite these efforts by the government, though, there has been no sustained progress in closing the equity gap or in reducing newborn mortality (Government of Nepal 2004a,b, 2010; Kc *et al*. 2011b; Daniels *et al.* 2013).

In 2006, Nepal introduced a safe delivery incentive programme (the Aama program), which aimed to increase institutional delivery (NHSSP 2009). Health facilities offered free delivery services as well as financial compensation for the mothers’ transport cost. The birth preparedness programme (BPP) to increase the knowledge, attitude and behaviour of community towards birth preparedness was started in 2007 (Powell-Jackson *et al*. 2009).

In 2007, the MoHP and the Department of Health Services devised an integrated package of newborn health interventions, called the Community Based Newborn Care Package (CB-NCP). This package has been described extensively elsewhere (Pradhan *et al*. 2011). Briefly, it comprised behavioural change communication for newborn care; promoted institutional delivery; postnatal home visits; community-based management of neonatal infections; and low birth-weight and birth asphyxia management (MoHP 2007). Although these interventions had been tested independently in different local and global contexts, they had never been evaluated as a package in the national health system setting of Nepal (Bhutta *et al*. 2005, 2008). The pilot implementation of this package was planned for 10 districts of the country and Saving Newborn Lives took initiative to support implementation and evaluation in the Bardiya district in 2009 (Pradhan *et al*. 2011).

An analysis was done of the coverage of postnatal home visits by trained cadres of community-based workers and volunteers using endline data from this evaluation of CB-NCP in Nepal and similar programs in Malawi and Bangladesh (Sitrin *et al.*, 2013). Half of the women interviewed in Nepal received a postnatal visit within 3 days after birth. Receiving a postnatal visit was found to be associated with FCHVs being linked with the mothers during pregnancy, notification by the family during labour/delivery, and having delivered outside a facility. An evaluation after the pilot phase of CB-NCP in Bardiya showed improvements ranging from 35 to 66% in coverage of some key indicators for care during pregnancy, during and after delivery (Pradhan *et al*. 2011).

### Current study aim

The evaluation by Pradhan *et al*. (2011) did not address the changes in equity.

Since improvements in overall newborn health indicators can mask persisting or widening inequalities (Kraft *et al*. 2013), we aimed to investigate whether there was a parallel improvement in equity in the indicators or uptake of healthy newborn care practices, as their percentage coverage increased.

## Materials and Methods

### Study setting

Bardiya district is situated in the mid-western region, southwest of Nepal’s capital city, Kathmandu. The district has a population of 459 141 with a population density of 227 per square kilometer. The district is administratively divided into one urban municipality area and 32 rural areas termed village development communities (VDCs). The VDCs are further divided into wards and Bardiya has a total of 288 wards.

Bardiya is one of the more disadvantaged districts in the mid-western region and it was selected for pilot implementation because it had high levels of inequality in access to services (World Bank and DFID 2006; Pradhan *et al*. 2011). In terms of the caste and ethnic group composition, about 50% of the population is lower caste Janjatis (considered disadvantaged), 15% Muslim and 34% higher caste Brahmin and Chettris. The district has one hospital, 3 primary health care centers, 8 health posts and 22 sub-health posts. At the time of the study there were a total of 216 facility-based health workers, 54 CHWs and 841 FCHVs. There were a total of 48 500 FCHVs around the country (Government of Nepal 2006; Ministry of Health and Population 2010). In Bardiya district, the average catchment population per FCHV was ∼546.

### Intervention and implementation in Bardiya

The implementation of the CB-NCP package has been described extensively elsewhere (Pradhan *et al*. 2011) and here we provide a brief summary of the process. Between March and December, 2009, all existing FCHVs, CHWs and facility-based health workers completed training and the components of this training are listed in Table 1. District-level planning for implementation was done through a multi-stakeholder consultation led by the district public health office. The stakeholders at district level were district health office staff, the district hospital superintendent, nursing staff, representatives of other line ministries such as the local development office and the district education office. At the village level, there was an orientation of the health facility operation committee to the package. This orientation emphasized the package’s potential impact on neonatal health. After the training of FCHVs, a MG meeting was organized to orient them to the new skills that FCHVs had acquired on newborn care. Similarly, there was an orientation of traditional birth attendants and traditional healers, emphasizing the importance of timely care-seeking for mothers and newborns.

**Table 1.** Components of the CB-NCP Training Package

Component	Description
Training of facility-based providers	A 7-day, competency-based training package for health workers focused on infection prevention, immediate and routine newborn care, assessment during the postnatal visit, assessment and management of newborns with infection, low birth weight, birth asphyxia and hypothermia as well as information and logistics management.
Training for CHWs (MCHWs/VHWs)	A 5-day training curricula focused on essential newborn care, assessment and management of infected newborns, low birth weight babies, referral management of sick newborn and information, logistics management and supervision support to FCHVs.
Training for FCHVs	A 6-day, competency-based training on social mobilization for birth preparedness, hand washing, clean delivery practices, essential newborn care, postnatal care, assessment and management of infections, low-birth weight, hypothermia and birth asphyxia and information management. The training also dealt with conducting MG orientation.
Equipping of health workers	Provision of equipment to facilities, CHWs, and FCHVs, including: thermometers, pan scales, de-lee suctions, bags and masks, salter scales, timers, clean delivery kit, tablet cotrim P, and job aids for all levels and materials for kangaroo mother care, injectable gentamycin, syringes, and disposal box for facilities and health posts operated by CHWs.

MCHW = Maternal Health Care Workers; VHW = Volunteer Health Workers.

The district health office encouraged monthly meetings between FCHVs and the health facilities-in-charge to ensure continuous support of the FCHVs by the facilities. FCHVs were given cash incentives that depended on the activities performed for CB-NCP (Pradhan *et al.*, 2011). Because this performance-based incentive scheme was linked with birth preparedness counselling as well as health institutional delivery, FCHVs increased their effort on counselling as well as accompanying the women to health facilities (Pradhan *et al*. 2011, 2012). In order to ensure regular knowledge and skill re-enforcement, on-site supportive supervision was provided to FCHVs and health workers. The FCHVs were trained to conduct four home visits during pregnancy and four home visits after delivery. They were provided with job aids developed by the BPP for counselling during their home visits. They were also encouraged to inform expectant mothers about the availability of incentives for antenatal attendance and facility delivery, and the removal of user fees for delivery at public health facilities through the MOH’s Aama programme (NHSSP 2009). Under this scheme, women in Bardiya district were given conditional cash transfers of 500 Nepali rupees if they delivered at a health facility, and an additional 400 Nepali rupees if they attended four antenatal check-ups.

Community mobilization and behaviour change activities included: (1) FM radio announcements of essential newborn messages; (2) street drama performances on newborn care messages by a professional art and music group ‘Surdaya Saskritik Partisthan’ (3) Billboards with newborn care messages; (4) television broadcasting at the Maternal Child Health clinic during clinic time; (5) FCHVs interacted with the community during a one-day social event, which was also broadcast live on the radio; (6) orientation of Health Facility Operation and Management committees, formal and informal political parties, social workers and teachers to the CB-NCP programme.

### Study design

This study was designed as a prospective before-and-after assessment which used baseline and endline survey data. The baseline household survey was conducted between June and November 2008. Full district-wide implementation was in place for 18 months between January 2010 and June 2011, and the endline survey was conducted, in July 2011. No comparison district was available.

The survey questionnaires included questions on knowledge of newborn danger signs, newborn care practices, and use of facility- and community-based maternal and newborn care services. Table 2 provides a description of the newborn indicators considered in this analysis. All respondents were informed of the survey purpose and procedures, and they provided oral consent. The Child Health Division and district authorities in Bardiya provided the permission for data collection.

**Table 2:** Outcome indicator variables and their descriptions

Indicator	Description
**Place of delivery**	Home vs facility (clinic, hospital, etc.)
**Recognize danger signs in newborn**	At least three danger signs among: fever, hypothermia, unable to breastfeed, umbilical pus/discharge, fast breathing (60 or more), severe chest-indrawing, unconsciousness/lethargy/less movement
**Newborn care**	
Nothing applied to cord	Nothing applied to cord immediately after cutting (home births only)
Immediate breastfeeding	Put to breast within 1 h of delivery
Delayed bathing	Bathing delayed for at least 6 h after birth
Baby dried	Baby dried before the placenta was delivered
**ANC/PNC visits**	
Antenatal care	Four or more antenatal care visits with any provider
1 ANC visit	At least one ANC visit with skilled provider
FCHV home visit during pregnancy	At least one home visit by FCHV during pregnancy
FCHV postnatal home visit within 3 days of delivery	At least one postnatal home visit by FCHV within 3 days of delivery
**Birth preparedness**	At least one of the following: identify HF/SBA, arrange transport and save money

### Sampling strategy

A two-stage cluster sampling approach was followed where 30 wards were selected with probability proportional to ward size. Within the selected wards, all households were screened to identify eligible respondents who were defined as women between 15 and 49 years who had delivered in the past 12 months. From each of these wards a random sample of 21 recently delivered mothers, for a total of 630 respondents, were sampled at baseline and endline.

### Statistical analysis

To assess the equity of the intervention activities and outcomes, we derived wealth scores from household socioeconomic status (SES) variables using principal components analysis (Filmer and Pritchett 2001; Vyas and Kumaranayake 2006). Based on these scores, households were divided into quartiles, from the most poor to the least poor. The SES variables included in the score were recorded in a similar manner at baseline and at endline, and they were availability of electricity; ownership of a bicycle, telephone, television, radio; source of drinking water; the type of toilet; materials of the floor, roof and walls. The outcome indicators considered were knowledge of newborn danger signs, newborn care practices, and use of facility- and community-based maternal and newborn care services, which are described in Table 2. All outcome indicators were coded as binary variables. We present the percentages of mothers who responded yes to each indicator by wealth quartile.

The wealth scores were used to plot the (CIndex) curves for each indicator (Wagstaff *et al*. 1991; Kakwani *et al.* 1997; O’Donnell *et al.*, 2008). These plots represent the cumulative proportions of the indicator variables vs cumulative proportions of participants as ranked by the wealth scores. Also included in each of these plots is the 45-degree line that indicates perfect equality where an indicator is equally distributed among participants across the wealth scores. The CIndex is derived from these curves as twice the area between the concentration curve and the line of perfect equality. We followed a regression approach for calculating this index and adjusted for clustering by wards (Kakwani *et al*. 1997). A CIndex equal to zero indicates equality of the indicator across the different wealth scores.

We conducted *t*-tests to compare the CIndex of each of the indicators between baseline and endline. A negative difference between baseline and endline indicated movement to a more equitable distribution of the health indicator across socioeconomic groups if the lower SES groups were under-represented at baseline, and vice versa. For the indicators which showed a statistically significant difference between baseline and endline, we also repeated the above analysis using ranking by education level, instead of ranking by wealth score. This was done because the endline sample had slightly more participants with more years of education.

We present results for mothers who reported live births in the year prior to the survey and excluded those who had stillbirths as they were not interviewed regarding newborn care practices and postnatal care.

## Results

Baseline characteristics of the participants are given in Table 3. There were 625 (out of 630) mothers at baseline and 615 (out of 630) at endline who had live births and were included in the analysis. The distributions of age, education, caste and religion were fairly balanced between baseline and endline samples. The only notable difference was a slightly higher percentage of women at baseline (54.6% vs 41.9%) who reported having no education. The majority of mothers in both samples were younger than 25, Hindu (>95%), and from the Terai Janajati caste (>62%). Following construction of the asset index for each survey, slightly more women (29.2%) were classified as being in the poorest quartile in the endline survey. The first principal components used for the asset indices accounted for 31% of variation in the asset variables in the baseline survey, and 25% in the endline survey.

**Table 3.** Participants’ characteristics

	Baseline (*n* = 625)	Endline (*n* = 615)	
	*n*(%)	*n*(%)	*P*-value (Chi-square/exact test for independence)
**Age-group**			
15–<20	95 (15.2)	96 (15.6)	
20–<25	282 (45.12)	284 (46.2)	
25–<30	154 (24.64)	169 (27.5)	
30+	94 (15.04)	66 (10.7)	0.14
**Education**			
None	341 (54.6)	258 (41.9)	
Primary	148 (23.7)	136 (22.1)	
Secondary	101 (16.2)	149 (24.2)	
SLC and above	35 (5.7)	72 (11.7)	<0.001
**Caste**			
TeraiJanjati	392 (62.7)	390 (63.4)	
Other*	233 (37.3)	225 (36.4)	0.80
**Religion**			
Hindu	611 (97.8)	586 (95.3)	
Other	14 (2.2)	29 (4.7)	0.017
**Asset quartiles**			
Poorest	162 (25.92)	181 (29.4)	
2	152 (24.32)	136 (22.1)	
3	161 (25.76)	145 (23.6)	
Least poor	150 (24.00)	153 (24.9)	

*Other castes: Brahamn/Chhetri, Tarai, Dalits, Newar and Muslim

Table 4 shows the values (in percentages) of newborn indicators overall and by wealth quartiles at baseline and endline, the corresponding mean concentration indices and their confidence intervals. The largest improvements in coverage, between baseline and endline across all wealth quintiles, were observed in facility delivery (31.5–81.5%), immediate drying (60.8–95.1%) and delayed bathing (66.6–95.4%). Data on FCHV home visits during pregnancy and after delivery were only available at endline. Nearly all (97.2%) mothers reported receiving at least one visit during pregnancy and this was equally distributed across all wealth quartiles [CIndex: −0.003 (−0.01, 0.0006)]. About half of mothers (49.9%) reported receiving a home visit for their newborn from an FCHV within 3 days of giving birth, with newborns in the poorest quartiles being more likely to receive a visit than the least poor [CIndex-0.06 (−0.12, 0.01)].

**Table 4.** Newborn indicators by wealth quartiles, corresponding concentration indices and their confidence intervals

**Variable**	**Baseline (%)**	**Endline(%)**	**Change in CIndex**
**Facility delivery**	***n* = 625**	***n* = 615**	
**Overall**	**31.5**	**81.5**	
Poorest	23.5	84.0	
Second	26.9	78.7	
Third	33.5	77.9	
Least poor	42.7	84.4	
Concentration index (95% CI)	0.16 (0.07,0.24)	0.006 (−0.02,0.03)	**−0.15 (−0.24, −0.06)**
**Recognize at least three danger signs**	***n* = 625**	***n* = 615**
**Overall**	**90.2**	**96.9**	
Poorest	84.6	97.2	
Second	89.5	96.3	
Third	91.3	97.2	
Least poor	96.0	96.7	
CIndex (95% CI)	0.026 (0.01, 0.05)	−0.001 (−0.01, 0.01)	**−0.026 (−0.05, −0.003)**
Nothing applied to cord*	***n* = 425**	***n* = 114**	
**Overall**	**70.0**	**86.0**	
Poorest	68.9	82.8	
Second	69.4	89.6	
Third	70.3	81.3	
Least poor	71.8	91.7	
CIndex (95% CI)	0.02 (−0.02,0.6)	0.04 (−0.19,0.28)	0.03 (−0.21,0.26)
**Breastfeed within 1 h**	*n* = 621**	***n* = 615**	
**Overall**	**64.3**	**89.6**	
Poorest	55.9	93.9	
Second	69.1	90.4	
Third	69.8	94.5	
Least poor	65.4	79.1	
CIndex (95% CI)	0.03 (−0.02, 0.08)	−0.03 (−0.05, −0.002)	**−0.05 (−0.11, −0.0001)**
**Immediate drying**	***n* = 625**	***n* = 615**	
**Overall**	**60.8**	**95.1**	
Poorest	63.6	95.6	
Second	65.1	95.6	
Third	59.0	94.2	
Least poor	55.3	94.1	
CIndex (95% CI)	−0.02 (−0.07, 0.03)	−0.005 (−0.02, 0.008)	0.02 (−0.03, 0.06)
**Delayed bathing**	***n* = 625**	***n* = 615**
**Overall**	**66.6**	**95.4**	
Poorest	**69.1**	97.2	
2^nd^	66.5	92.7	
3^rd^	65.8	93.85	
Least poor	64.7	97.4	
CIndex (95% CI)	0.02 (−0.01, 0.05)	0.003 (−0.01, 0.01)	−0.02 (−0.04, 0.01)
**At least one ANC visit with skilled provider**	***n* = 625**	***n* = 615**	
**Overall**	**55.0**	**65.5**	
Poorest	44.4	68.5	
Second	50	52.9	
Third	57.1	64.1	
Least poor	69.3	74.5	
CIndex(95% CI)	0.025 (0.01, 0.04)	0.000009 (−0.0002, 0.0002)	**−0.025 (−0.04, −0.01)**
**At least four ANC visits**	***n* = 625**	***n* = 615**	
**Overall**	**57.8**	**81.1**	
Poorest	42.0	84.5	
Second	51.3	77.2	
Third	59.6	76.6	
Least poor	79.3	85.0	
CIndex (95% CI)	0.15 (0.11, 0.18)	0.002 (−0.03, 0.03)	**−0.15 (−0.19, −0.10)**
**At least one FCHV home visit during pregnancy**		***n* = 615**	
**Overall**		**97.2**	
Poorest		97.2	
Second		98.5	
Third		97.9	
Least poor		95.4	
CIndex (95% CI)		−0.003 (−0.01, 0.006)	
**At least one FCHV home visit within 3 days after birth**		***n* = 615**	
**Overall**		**49.9**	
Poorest		56.9	
Second		52.9	
Third		48.3	
Least poor		40.5	
CIndex (95% CI)		−0.06 (−0.12, 0.01)	
**Birth preparedness**	***n* = 625**	***n* = 615**
**Overall**	**70.2**	**90.1**	
Poorest	50.0	86.2	
Second	71.7	89.7	
Third	74.5	89.7	
Least poor	86.0	95.4	
CIndex (95%CI)	0.12 (0.08,0.15)	0.02 (0.01,0.04)	**−0.09 (−0.12, −0.06)**

*The cord care indicator was only collected among mothers who delivered at home

**4 missing responses

The 95% confidence intervals of the tests for the differences in CIindex from baseline to endline indicated that there was a significant improvement in the equity of facility delivery [−0.15 (−0.24, −0.06)], recognizing at least three newborn danger signs [−0.026 (−0.05, −0.003)], immediate breastfeeding [−0.05 (−0.11, −0.0001)], at least one ANC visit with a skilled provider [−0.025 (−0.04, −0.01)], at least four ANC visits from any provider [−0.15 (−0.19, −0.10)], and birth preparedness [−0.09 (−0.12, −0.06)]. Reported prevalence of other newborn care practices improved but their equity across wealth quartiles did not improve significantly.

We conducted a sensitivity sub-analysis to determine whether ranking participants by levels of education rather than by household assets confirmed the significant differences in equity found when using asset scores. The significant differences in equity were maintained as follows: facility delivery [CIndex: −0.312 (−0.389, −0.234)], knowledge of at least three newborn danger signs [−0.037 (−0.06, −0.015)], breastfeeding within 1 h [−0.157 (−0.209, −0.104)], at least one antenatal visit with a skilled provider [−0.039 (−0.056, −0.022)], at least four antenatal visits from any provider [−0.197 (−0.242, −0.153)] and birth preparedness [−0.138 (−0.184, −0.093)].

## Discussion

The CB-NCP was developed to improve neonatal survival by increasing the utilization of routine health services such as antenatal care, skilled attendance at birth, postnatal care and community-based care of sick newborns. It also aimed to improve essential newborn care and care-seeking behaviour. Strengthening services at health facilities were also a key focus of CB-NCP as health workers were trained in managing referred sick newborns, managing low birth-weight babies and those who had suffered birth asphyxia. In this analysis, we investigated the changes in equity of some of the key maternal and newborn care indicators and also presented the changes in coverage of these indicators.

We saw significant changes in equity for facility delivery, recognizing three newborn danger signs, breastfeeding within an hour and ANC visits with a skilled provider. The proportion of women delivering at facilities more than doubled over the 2.5 years between baseline and endline. This is attributed to the effects of the incentive-based Aama program, because in a wider multi-district assessment of CB-NCP districts, there were also marked increases in institutional deliveries, and they were of the same magnitude as in non-CB-NCP districts (McPherson 2013).

We also saw increases in coverage of antenatal and postnatal care visits, improved birth preparedness, newborn-care practices that included proper cord care, immediate breastfeeding and drying and delayed bathing.

We observed a significant difference in the distribution of education status among participants at baseline compared with those at endline. Education levels and household asset scores are correlated as, in general, an increase in education leads to an increase in asset possession. Hence, it is common practice to include analysis of education level-based inequalities alongside wealth score-based ones (McKinnon *et al*. 2014). We thus conducted a parallel analysis to determine whether there was an equitable distribution of the intervention indicators when participants were ranked by education level rather than by household asset index. This confirmed the significant improvements in equity for the same indicators, namely, delivery place; knowledge of at least three newborn danger signs; at least one antenatal visit with a skilled provider; at least four antenatal visits from any provider; and birth preparedness. The improvements in equity over wealth score rankings were similar to the improvements over education level ranking. Thus, at endline, the participants with no education or lower levels of education had a fairer share of the distribution of these newborn care practices than their counterparts at baseline. Our results are similar to those found by a meta-analysis of McKinnon *et al.* (2014) which showed overall decrease in wealth-based and education-based inequalities in neonatal mortality rates over time.

A key intervention to improve newborn child survival which is also highlighted by the 2009 WHO-UNICEF joint statement on home visits is ‘a home visit within 3 days of birth in settings where most of deliveries take place at home’ (WHO 2009). In our evaluation, we found that there was inequity in this indicator as 40.5% of the least poor received this visit as opposed to 56.9% of the poorest. This suggests that there may have been bias in favour of the poorest and that further efforts are needed to better link newborns with community-based postnatal services by FCHVs, irrespective of SES.

Our results were similar to those of the community-based mobilization programmes found in similarly low-resource settings that indicate that these programs tend to improve usage of healthcare facilities and newborn care behaviours (Baqui *et al*. 2008b; Barros *et al.* 2012a; Kumar *et al*. 2012; Victora *et al*. 2012; Malqvist *et al*. 2013). A quasi-experimental study in India by an NGO showed improvements in equity in health care utilization for mothers and newborns in the intervention district, but notable socioeconomic differentials remained. Improvements in equity were mostly pronounced for household practices. Overall programme coverage remained low and that limited the ability to address inequity (Baqui *et al*. 2008b).

Our findings are also similar to those of a cluster-randomized controlled trial in Shivgarh, India which showed significant improvements in equity of indicators including knowledge of danger signs, care practices, self-reported complications, and timely care seeking from trained providers (Kumar *et al*. 2012).

An equity evaluation was done after an implementation of a similar package in Malawi called the Community-Based Maternal and Newborn Care (CBMNC). The context in Malawi was similar to that of CB-NCP in Nepal in that the programme was implemented within the country’s health system where other maternal and newborn care programmes were already in place. Thus improvements in coverage and equity could not be attributed to CBMNC alone. The results showed improved coverage (though modest) and equity in the knowledge of danger signs for both maternal and newborn health. There were also moderate to high levels of facility delivery, delayed bathing and immediate breastfeeding, and equity in this was improved (Callaghan-Koru *et al*. 2013). Equity improvements in Nepal were much larger than those observed in Malawi. This was possibly due to the combination of incentives and the greater reach of the CB-NCP Nepal package compared with the CBMNC Malawi package. The latter only reached a small proportion of mothers and newborns compared with what we observed in Nepal.

## Programme strengths and limitations

Under CB-NCP, the systematic efforts from the district health office, local health facilities and community groups helped to create an enabling environment for FCHVs to promote birth preparedness and institutional delivery, to emphasize the importance of antenatal care and to conduct home visits for maternal and newborn care (Pradhan *et al*. 2011). This programme was implemented alongside already-existing incentive-based programs that helped generate demand and mediate financial barriers to seeking care.

Packages similar to CB-NCP tested in controlled trial settings resulted in reduction in neonatal mortality; however, when implemented within a country’s health system through routine workers, they showed some improvement in health care utilization while inequity in accessing the services remained the same (Haws *et al.* 2007; Baqui *et al*. 2008a; Lassi *et al*. 2010). In comparison, the use of this package in Nepal, with the help of FCHVs, contributed to the improvement in coverage and equity of healthcare utilization.

This was a before- and after-evaluation with no comparison area. We were unable to control for confounding factors of other health programmes. As a result, this evaluation provides only an adequacy assessment (Habicht and Pelto 2014) and we are unable to attribute the improvement of the health indicators to the effect of CB-NCP on its own. Attributing changes to a specific programme is increasingly challenging in low-income country settings, where more partners are acting to improve public health at the same time (Victora *et al*. 2011).

A minor limitation is that a small subset of mothers (2.4% at baseline and 0.8% at endline) had stillbirths and were thus not interviewed. Such mothers may have had different experiences than those who had livebirths. Given that this was only a very small subset of mothers, we believe that our results are unlikely to be biased by the lack of their responses.

## Conclusion and recommendation

Nepal is one of a few countries reaching the MDG for child survival due to the accelerated reduction in post-neonatal mortality. However, a similar decline in neonatal mortality is desperately needed. The community-based neonatal care packages have been shown to improve utilization and equity of maternal and neonatal care services. We recommend that these programs be continually evaluated and improved in order to make it feasible for them to be scaled up to the national level. We also recommend that equity be evaluated in other settings within Nepal in order to determine if there is improvement in those areas as well. This should be done keeping in mind that the extent of the improvements in equity seen in Bardiya may not be replicated in other areas because Bardiya had exceptionally high levels of inequity at baseline, compared with other areas, and received additional support for programme implementation from an NGO partner.

## Ethical considerations

The programme was implementing national policy through the routine system. To operationalize the National Neonatal Health Strategy, the Nepal Ministry of Health and Planning initiated the development of the Community-Based Newborn Care Package, which outlined the role of Save the Children in supporting the government to develop and test the package. Data collection was completed as part of routine programmatic activities. Relevant district authorities granted permission and all respondents provided oral consent upon being informed of the purpose of data collection. Consent was documented by interviewers on the questionnaires.
